# Cerebral embolic protection during transcatheter aortic valve replacement: a systematic review and meta-analysis of propensity score matched and randomized controlled trials using the Sentinel cerebral embolic protection device

**DOI:** 10.1186/s12872-023-03338-0

**Published:** 2023-06-17

**Authors:** Mathias Wolfrum, Immanuel Justus Handerer, Federico Moccetti, Alexander Schmeisser, Ruediger C. Braun-Dullaeus, Stefan Toggweiler

**Affiliations:** 1 Heart Center Lucerne, Luzerner Kantonsspital, Lucerne, Switzerland; 2 Department of Internal Medicine, Division of Cardiology and Angiology, Magdeburg University, Magdeburg, Germany

**Keywords:** Aortic stenosis, TAVR, Transcatheter aortic valve replacement, Cerebral protection, Outcomes

## Abstract

**Background:**

The Sentinel cerebral embolic protection device (CEP) aims to reduce the risk of stroke during transcatheter aortic valve replacement (TAVR). We performed a systematic review and meta-analysis of propensity score matched (PSM) and randomized controlled trials (RCT) investigating the effect of the Sentinel CEP to prevent strokes during TAVR.

**Methods:**

Eligible trials were searched through PubMed, ISI Web of science databases, Cochrane database, and proceedings of major congresses. Primary outcome was stroke. Secondary outcomes included all-cause mortality, major or life-threatening bleeding, major vascular complications and acute kidney injury at discharge. Fixed and random effect models were used to calculate the pooled risk ratio (RR) with 95% confidence intervals (CI) and absolute risk difference (ARD).

**Results:**

A total of 4066 patients from 4 RCTs (3′506 patients) and 1 PSM study (560 patients) were included. Use of Sentinel CEP was successful in 92% of patients and was associated with a significantly lower risk of stroke (RR: 0.67, 95% CI: 0.48–0.95, *p* = 0.02. ARD: -1.3%, 95% CI: -2.3 – -0.2, *p* = 0.02, number needed to treat (NNT) = 77), and a reduced risk of disabling stroke (RR: 0.33, 95% CI: 0.17–0.65. ARD: -0.9%, 95% CI: -1.5 – -0.3, *p* = 0.004, NNT = 111). Use of Sentinel CEP was associated with a lower risk of major or life-threatening bleeding (RR: 0.37, 95% CI: 0.16–0.87, *p* = 0.02). Risk for nondisabling stroke (RR: 0.93, 95% CI: 0.62–1.40, *p* = 0.73), all-cause mortality (RR: 0.70, 95% CI: 0.35–1.40, *p* = 0.31), major vascular complications (RR: 0.74, 95% CI: 0.33–1.67, *p* = 0.47) and acute kidney injury (RR: 0.74, 95% CI: 0.37–1.50, *p* = 0.40) were similar.

**Conclusions:**

The use of CEP during TAVR was associated with lower risks of any stroke and disabling stroke with an NNT of 77 and 111, respectively.

**Supplementary Information:**

The online version contains supplementary material available at 10.1186/s12872-023-03338-0.

## Introduction

If left untreated, aortic stenosis is associated with a high risk of morbidity and mortality [1, 2]. Transcatheter aortic valve replacement (TAVR) is an establishedtreatment for aortic stenosis and has been extensively investigated in randomized controlled trials (RCT) in the whole spectrum of surgical risk categories. TAVR has demonstrated excellent short- and mid-term safety and efficacy, compared to surgical aortic valve replacement (SAVR) [1–7]. However, overt stroke is still the most devastating complication of TAVR, occurring in 0.6 to 5% of patients during early follow-up [1–7]. Stroke is associated with high morbidity and mortality, and costs [8–10].

If TAVR is considered for use in younger patients with aortic stenosis and low surgical risk, the rate of stroke has to be minimised. Several cerebral embolic protection (CEP) devices have been developed. Some data from observational studies, randomized controlled trials and ultimately from meta-analysis suggest that the use of CEP devices in general was associated with a lower risk of stroke [11, 12]. Contrary, although CEP devices seem to reduce lesion volume during TAVR, this effect did not translate into a reduction in hard clinical outcomes as shown by another meta-analysis [13]

However, only the Sentinel CEP is approved by the FDA and readily available for cerebral protection during TAVR. The Sentinel CEP device is a dual-filter embolic protection device which is implemented percutaneously through a ﻿6 French trans-arterial catheter prior to TAVR. The two embolic filters are placed: one in the brachiocephalic artery (proximal filter) and one in the left common carotid artery (distal filter). The filters are removed from the patient after completion of the intervention [14]. The recent PROTECTED TAVR trial randomized 3000 patients to TAVR either with or without the Sentinel CEP device. The rate of stroke after TAVR through 72 h, or at discharge, was numerically lower in patients treated with the CEP device. However, due to the low number of primary events and the low effect size, the trial was deemed underpowered. Therefore, the present meta-analysis reviews data from all available propensity score matched and randomised controlled trials using the Sentinel CEP device to further investigate its effect on stroke and other clinical outcomes in a larger cohort.

## Methods

﻿We performed a systematic review and study-level meta-analysis of propensity score matched (PSM) studies and RCTs that tested the efficacy of Sentinel CEP device during TAVR according to current recommendations of the Cochrane Handbook for Systematic Reviews and the Meta-analysis of Interventions [15] and in compliance with the PRISMA (Preferred Reporting Items for Systematic reviews and Meta-Analyses) statement in healthcare interventions [16]. The meta-analysis was registered in the Open-Science-Framework registry (OSF. May 18. osf.io/jw9bz).

### Study design and endpoints

PSM studies and RCTs investigating the efficacy of the Sentinel CEP device during TAVR were included. All other studies reporting outcomes with this CEP device were excluded, in order to reduce the selection and confounding biases of those studies. Two groups were compared, defined by intraprocedural use or no use of the Sentinel CEP device. The pre-specified primary outcome was stroke (all-cause stroke, disabling stroke, nondisabling stroke). Secondary endpoints included all-cause and cardiovascular mortality, major vascular complications, major or life-threatening bleeding, acute kidney injury at discharge or if unavailable at 30 days. Outcomes were defined according to the Valve Academic Research Consortium – 2 (VARC-2) or Neurologic Academic Research Consortium (NeuroARC) definitions [17, 18]. All endpoints were estimated according to the intention-to-treat (ITT) principle.

### Search strategy

PubMed, Cochrane Library, ISI Web of science databases, and TCTMD.org were searched for abstracts, papers, and conference reports published up to September 20, 2022. The references of the selected articles were checked for other relevant citations. There were no language restrictions. The following combination of key words and Medical Subject Heading (MeSH) terms were used: “cerebral embolic protection,” “Sentinel cerebral protection system,” “transcatheter aortic valve implantation,” and “transcatheter aortic valve replacement” (Online Table S1 details search strategy for PubMed). Two authors (M.W. and I.H.) independently identified appropriate articles. Disagreements regarding study selection were resolved by consensus by a third independent investigator (F.M.)

### Data extraction

Pre-determined data elements were extracted from each trial and entered in a structured dataset, including: baseline characteristics, TAVR access site, type of THV, clinical outcome measures (stroke, all-cause mortality, major/life-threatening bleeding, major vascular complications, acute kidney injury), and device success (defined as the correct deployment and retrieval of the device).

### Data synthesis and analysis

Pooled risk ratios and 95% confidence intervals (CI) for binary outcomes were calculated. As mostly rare events were encountered, the Mantel–Haenszel method, which can include single-zero and double-zero studies, was used to combine the results [19]. The decision between fixed effect or random effect model was based on the characteristics of the individual studies. As no relevant differences in comorbidities of enrolled patients or regarding procedure techniques were identified the fixed effect model was primarily used in our analysis [15]. Furthermore, heterogeneity among trials was estimated and displayed for each outcome using chi-square tests and quantified with *I*^2^ statistics. Heterogeneity was considered low for *I*^2^ < 25%, moderate for *I*^2^ < 75%, and high for *I*^2^ ≥ 75% [20]. Only for the endpoint of major vascular complications moderate heterogeneity was found, subsequently the random effect model was used for this endpoint. Publication bias for each outcome was estimated via visual inspection of the funnel plot. All analyses were conducted with the STATA statistical software package (Version 16.1, StataCorp, College Station, Texas, USA) and Review Manager (RevMan, Version 5.4, The Cochrane Collaboration, 2020).

## Results

### Included studies

**Fig. 1:**
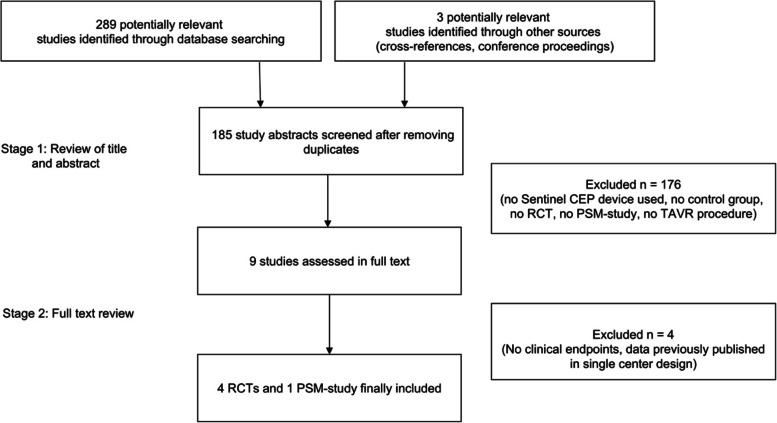
Study selection process. CEP, cerebral embolic protection. M-H. RCT, randomized controlled trial. PSM, propensity score matched. TAVR, transcatheter aortic valve replacement

A total of 5 studies with 4066 patients met our inclusion criteria: four randomized controlled trials (3′506 patients) and one propensity score matched study (560 patients), published between 2016–2022 (Fig. 1). Study characteristics are presented in Table 1. Overall risk of bias was deemed low in all 5 trials.

**Table 1:** Study characteristics

	Study name (Reference)				
	CLEAN-TAVI [21]	MISTRAL-C [22]	SENTINEL [14]	Seeger et al. [23]	PROTECTED TAVR [24]
Study design	RCT, single-center	RCT, multicenter	RCT, multicenter	PSM, single-center	RCT, multicenter
Year of publication	2016	2016	2017	2017	2022
Patients (n) ITT	100	65	363	280	3000
Age, years	80	82	83	81	79
Female sex	14%	6%	29%	15%	40%
Hypertension	91%	68%	NA	NA	87%
Diabetes	48%	20%	35%	30%	34%
Atrial fibrilation	34%	25%	24%	36%	33%
Prior stroke	4%	NA	6%	9%	8%
PAVD	6%	31%	15%	26%	11%
STS PROM score	5.4%	4.8%	6.0%	6.5%	3.4%
Valve type	CoreValve: 100%	SAPIEN 3: 54% CoreValve: 25% SAPIEN XT: 15% Others: 6%	SAPIEN 3: 52% Evolut R: 26% SAPIEN XT: 18% CoreValve: 4%	SAPIEN 3: 60% Lotus: 28% Evolut R: 12%	SAPIEN 3: 64% Evolut R/Evolut PRO: 25% Acurate: 7% Portico: 3% Lotus: 1%
TAVR route	transfemoral: 100%	Transfemoral: 100%	transfemoral: 95%	Transfemoral: 100%	Transfemoral: 100%
Sentinel device success	92%	94%	94%	92%	98%
Primary Outcome	Imaging: new cerebral lesions (DW-MRI)	Imaging: new cerebral lesions (DW-MRI)	Safety: MACCE (VARC-2) Efficacy: new cerebral lesions (DW-MRI)	death or stroke	stroke
Stroke definition	VARC-2	VARC-2	VARC-2	VARC-2	NeuroARC
Stroke assessment	Blinded, consecutive neurologic assessment plus imaging	Blinded, consecutive neurologic assessment plus imaging	Blinded, consecutive neurologic assessment plus imaging	Blinded, consecutive neurologic assessment, MRI if stroke was suspected	consecutive neurologic assessment, imaging if stroke was suspected

*CAD* coronary artery disease, *ITT* intention to treat, *MACCE* major adverse cardiac and cerebrovascular events, *NA* not available, *NeuroARC* Neurologic Academic Research Consortium, *PSM* propensity score matched, *RCT* randomised controlled trial, *STS* society of thoracic surgeons, *VARC-2* Valve Academic Research Consortium-2

### Primary endpoint

#### Stroke

**Fig. 2:**
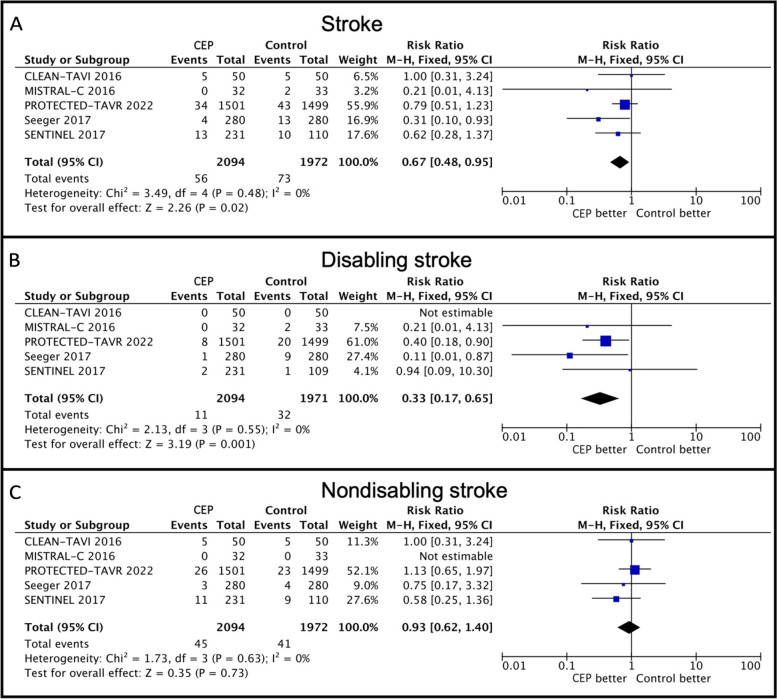
Stroke in patients undergoing TAVR with versus without Sentinel CEP. Forest plots of individual and summarized risk ratios of all-cause stroke (**A**), disabling stoke (**B**), nondisabling stroke (**C**) according to the use of the Sentinel CEP device versus not during TAVR. CI, confidence interval. CLEAN-TAVI, Claret Embolic Protection and TAVI. CEP, cerebral embolic protection. M-H, Mantel–Haenszel. MISTRAL-C, MRI Investigation With Claret. TAVR, transcatheter aortic valve replacement

All 5 trials contributed to the analysis of stroke events, with 4066 patients included (Fig. 2, Panel A). Patients in the Sentinel CEP device group had a lower risk of stroke compared with the control group (2.7% vs. 3.7%. RR: 0.67, 95% CI: 0.48–0.95, *p* = 0.02, heterogeneity *p* = 0.48, *I*^2^ = 0%). Absolute risk difference (ARD) was -1.3% (95% CI: -2.3 – -0.2, *p* = 0.02, heterogeneity *p* = 0.29, *I*^2^ = 20%, number needed to treat (NNT) = 77).

#### Disabling stroke

All 5 trials reported on the rate of disabling stroke events, with 4065 patients included in the analysis (Fig. 2, Panel B). Patients in the Sentinel CEP group had a lower risk of disabling stroke compared with the control group (0.5% vs. 1.6%. RR: 0.33, 95% CI: 0.17–0.65, *p* = 0.001, heterogeneity *p* = 0.55, *I*^2^ = 0%). ARD was -0.9% (95% CI: -1.5 – -0.3, *p* = 0.004, heterogeneity *p* = 0.24, *I*^2^ = 0%, NNT = 111).

#### Nondisabling stroke

All 5 trials contributed to the analysis of nondisabling stroke events, with 4066 patients included in the analysis (Fig. 2, Panel C). No difference in risk of nondisabling stroke was found between patients in the Sentinel CEP and control group (2.1% vs. 2.1%. RR: 0.93, 95% CI: 0.62–1.40, *p* = 0.73, heterogeneity *p* = 0.63, *I*^2^ = 0%).

### Secondary endpoints

#### All-cause mortality

**Fig. 3:**
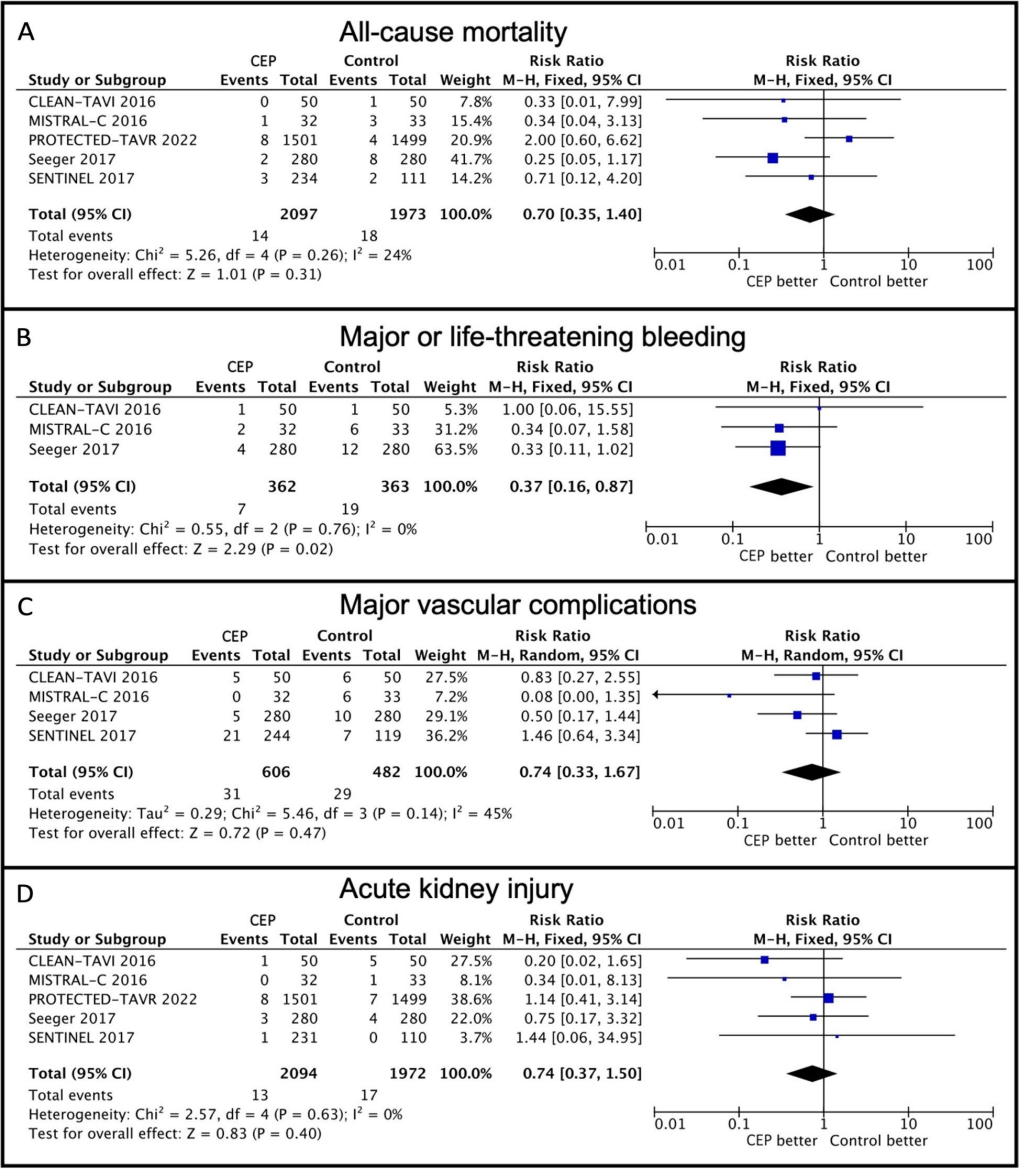
Secondary clinical outcomes in patients undergoing TAVR with versus without Sentinel CEP. Forest plots of individual and summarized risk ratios of all-cause mortality (**A**), major or life-threatening bleeding (**B**), major vascular complications (**C**) and acute kidney injury (**D**) according to the use of the Sentinel CEP device versus not during TAVR. CI, confidence interval. CLEAN-TAVI, Claret Embolic Protection and TAVI. CEP, cerebral embolic protection system. M-H, Mantel–Haenszel. MISTRAL-C, MRI Investigation With Claret. TAVR, transcatheter aortic valve replacement

All 5 trials contributed to the analysis of all-cause mortality, with 4066 patients included (Fig. 3, Panel A). No significant difference in risk of death was found between patients in the Sentinel CEP and control group (0.7% vs. 0.9%. RR: 0.70, 95% CI: 0.35–1.40, *p* = 0.31, heterogeneity *p* = 0.26, *I*^2^ = 24%).

#### Major or life-threatening bleeding

3 trials reported on the rate of major or life-threatening bleeding, with 725 patients included in the analysis (Fig. 3, Panel B). Patients in the Sentinel CEP group had a lower risk of major or life-threatening bleeding compared with the control group (1.9% vs. 5.5%. RR: 0.37, 95% CI: 0.16–0.87, *p* = 0.02, heterogeneity *p* = 0.76, I^2^ = 0%). ARD was -3% (95% CI: -6.0 – -1.0, *p* = 0.02, heterogeneity *p* = 0.27, I^2^ = 24%).

#### Major vascular complications

4 trials reported on the rate of major vascular complications, with 1088 patients included in the analysis (Fig. 3, Panel C). No significant difference in risk of major vascular complications was found between patients in the Sentinel CEP and control group (5.1% vs. 6.0%. RR: 0.74, 95% CI: 0.33–1.67, *p* = 0.47, heterogeneity *p* = 0.14, *I*^2^ = 45%, random effects model).

#### Acute kidney injury

All 5 trials contributed to the analysis of acute kidney injury, with 4066 patients included (Fig. 3, Panel D). No significant difference in risk of acute kidney injury was found between patients in the Sentinel CEP and control group (0.6% vs. 0.9%. RR: 0.74, 95% CI: 0.37–1.50, *p* = 0.40, heterogeneity *p* = 0.63, *I*^2^ = 0%).

## Discussion

Our meta-analysis included all available propensity score-matched and randomized clinical trial data, with the use of the Sentinel CEP device in patients undergoing TAVR. The results suggest that use of the Sentinel CEP effected a significant reduction in the risk of all-cause and disabling stroke. The CEP device can be used in most patients (procedural success > 90%) with a good safety profile, i.e., no increase in the risk of major vascular complications or acute kidney injury.

Cerebrovascular events continue to be a major complication associated with TAVR during short-term [25] and long-term follow up [26]. As stroke after TAVR is a relevant predictor for morbidity and mortality [27], reducing the risk of stroke is an important goal, especially when considering patients who are relatively young and have low surgical risk profile. Interest in neuroprotection during TAVR has led to the development of several types of CEP devices in order to reduce thrombembolic events during valve implantation procedures [28]. The Sentinel CEP device is the only FDA approved system. Previous studies have consistently demonstrated that the Sentinel CEP device captures debris in up to 99% of patients [28]. Imaging studies showed that patients treated using the Sentinel CEP had fewer new brain lesions, lower total lesion volume [22], and reduced frequency of ischemic cerebral lesions in potentially protected areas [21] than patients treated without the device. Some data suggest that use of the Sentinel CEP device has the potential to preserve early neurocognitive performance after TAVR [22].

### Debate on stroke

Recently, the large scale PROTECTED TAVR RCT reported data investigating the effect of the Sentinel CEP on the important endpoint of procedure-related stroke in patients undergoing TAVR [24]. The primary endpoint of stroke within 72 h after TAVR or before discharge did not significantly differ between the CEP and the control group (2.3% vs. 2.9%, *p* = 0.30). While the incidence of stroke in the CEP group was in the anticipated range, the number of events in the control group was lower than expected during the planning phase of the trial. The trial was statistically underpowered to detected such a small effect of the Sentinel CEP device. The low event rate in the control group is a good sign, as it demonstrates progress with the contemporary TAVR procedure in relation to strokes, even without the use of a CEP device. However, patients enrolled in the PROTECTED TAVR study had also a lower surgical risk than those in previous studies, potentially explaining the low stroke rate in the control group [14, 21–23]. Adding data from other studies our meta-analysis suggest a significant lower risk for all-cause stroke with the use of the Sentinel CEP device during TAVR (RR: 0.67, ARD -1.3%).

The incidence of disabling stroke is of outmost importance from the perspective of both patients and clinicians. Our meta-analysis suggests a significant lower risk of disabling stroke with the use of the Sentinel CEP compared to the control group (RR: 0.33, ARD -0.9%, NNT 111). This is in line with the PROTECT TAVR trial, which found a significantly lower rate of disabling stroke in the Sentinel CEP group compared to control group (0.5% vs. 1.3%) [24]. Based on these data, one additional disabling stroke could be prevented for every 125 patients treated with the Sentinel CEP device.

Interestingly, neither our meta-analysis, nor any single trial among those analyzed, found that use of the Sentinel CEP device was effective at preventing nondisabling strokes during TAVR. Reasons for this are not entirely clear, but the design of the Sentinel CEP device provides one possible explanation: The pore size of the filters (140 µm) blocks large, but not small emboli, potentially preventing larger disabling strokes, rather than smaller non-disabling strokes. However, the fact that blockage of small cerebral arteries can lead to clinical meaningful strokes, depending on the brain area does question the former theory. A clear answer why the CEP device does not influence non-diabaling strokes cannot be given from out analysis.

### When should the Sentinel CEP device be used during TAVR?

Based on results of our meta-analysis, the Sentinel CEP device shows an excellent safety profile and potentially prevents periprocedural strokes. The number needed to treat to prevent one stroke according to our analysis is about 77 patients. Assuming a price per CEP device of about $2′000 would translate into expected total costs of about $154′000 to prevent one stroke. However, lifetime cost per person after stoke is with $103′576 also substantial [29]. In which population the use of Sentinel CEP device is cost-effective remains uncertain. It might be a valid strategy to use the CEP device in a younger TAVR population but refrain from using it in the very eldery (≥ 85–90 years of age).

Overall our results might encourage some operators to continue to use the Sentinel CEP device. If only the trial results from the recent large PROTECTED TAVR trial with the missed primary endpoint are considered, however, some physicians will refrain from using the CEP device to avoid adding relevant cost to the TAVR procedure without a proven clinical benefit. Furthermore, it is not entirely clear who might benefit from the use of CEP devices, as no direct link has been established between the amount of captured debris, anatomical/clinical factors, and the rate of stroke [27, 30, 31]. The recent PROTECTED TAVR trial failed to identify a subgroup of patients that might particularly benefit from the CEP device [24]. We recommend a careful discussion between physicians and patients regarding the use of the Sentinel CEP, incorporating the risks and benefits based on our meta-analysis and other data from recent randomized trials.

We are eagerly awaiting the large BHF PROTECT TAVI study, which will enroll 8000 patients and provide more conclusive information to the interventional community about the effectiveness of the Sentinel CEPS during TAVR. But we must be cautious. Assuming a similar rate of stroke for the awaited BHS PROTECT TAVI trial as seen in the recently published PROTECTED TAVR trial the BHS PROTECT TAVI trial might still fail to demonstrate a protective effect of the Sentinel CEP device. 22′084 patients would be required for an adequately powered study to give a 80% chance of detecting a significant reduction in stroke, assuming an incidence of 2.3% in the Sentinel CEP group vs. 2.9% in the control group as found in the PROTECTED TAVR trial [24].

### Limitations

The number of available studies included in our meta-analysis was limited. Except for PROTECTED TAVR, the studies had small sample sizes, considering the very low event rate of the primary outcome. This meta-analysis used study level data, as we were unable to access patient-level data. The assessment of stroke varied among the trials, from just clinical assessment to thorough diagnostic workup by neurologists in conjunction with imaging studies. These circumstances might have led to under-detection or over-detection of events. Furthermore, the events were not always adjudicated by an independent clinical events committee.

Some studies did not report on the sites of bleeding, and therefore, the reason for lower bleeding events in cases using the Sentinel CEP remains unclear. Finally, data regarding other important outcomes, such as intra-procedural stroke, cost, length of hospital stay, re-admission, and longer-term outcomes were scarce in the individual trials and could not be incorporated in our meta-analysis. Sensitivity analysis considering only randomized controlled trials demonstrated a trend towards a lower rate of stroke with the use of the Sentinal CEP device [RR 0.76, (95% CI: 0.52, 1.09), I^2^ = 0%]. Incorporating propensity score-matched observational data might have overestimated the effect size of the CEP device.

## Conclusion

Results of our meta-analysis of clinical trial data for patients undergoing TAVR suggest a significant reduction in the risk of stroke with the use of the Sentinel CEP device. The device can be used in most patients and has a good safety profile, showing no increase in the risk of major vascular complications or acute kidney injury. Larger randomized clinical trials are warranted to confirm these results. The large BHF PROTECT TAVI study should further define the effectiveness of the Sentinel CEP in preventing strokes during TAVR.

## Supplementary Information

**Additional file 1.**

## Data Availability

The datasets used and/or analysed during the current study are available from the corresponding author on reasonable request.

## Abbreviations

CEP: Cerebral embolic protection
TAVR: Transcatheter aortic valve replacement
THV: Transcatheter heart valve
