# Supplementary material for: Cerebral embolic protection during transcatheter aortic valve replacement: a systematic review and meta-analysis of propensity score matched and randomized controlled trials using the Sentinel cerebral embolic protection device
Source: BMC Cardiovasc Disord. 2023 Jun 17;23:306. doi: 10.1186/s12872-023-03338-0 (PMC10276451; doi:10.1186/s12872-023-03338-0)
Supplement: Supplementary file 1 — Additional file 1. [file 12872_2023_3338_MOESM1_ESM.docx]

**SUPPLEMENT TO: Cerebral embolic protection during transcatheter aortic valve replacement: a systematic review and meta-analysis of propensity score matched and randomized controlled trials using the Sentinel cerebral embolic protection device.**

**SUPPLEMENTAL TABLES**

**Online Table S1.** Search strategy for Pubmed (search date September 20, 2022).

| **Search** | **# of abstracts** |
| --- | --- |

Search History

Limits: no

#5 Search (#1 OR #2) AND (#3 OR #4) 125

#4 Search “transcatheter aortic valve replacement” [All] 12349

#3 Search " transcatheter aortic valve implantation" [All] 6991

#2 Search " Sentinel cerebral protection system " [All] 25

#1 Search " cerebral embolic protection " [All] 186
